# Cutaneous metastasis of carcinomatous component of ovarian carcinosarcoma: A case report and review of the literature

**DOI:** 10.1186/s13000-022-01256-x

**Published:** 2022-10-05

**Authors:** Jinhang Li, Chen Cao, Peng Liu, Zhifeng Yan, Deyin Xing, Aijun Liu

**Affiliations:** 1 Department of Pathology, The First Medical Center of PLA General Hospital, Beijing, China; 2 Department of Obstetrics and Gynecology, The Seventh Medical Center of PLA General Hospital, Beijing, China; 3 Department of Pathology, The Johns Hopkins University School of Medicine, 21231 Baltimore, MD USA; 4 Department of Pathology, The Seventh Medical Center of PLA General Hospital, 100700 Beijing, China

**Keywords:** Cutaneous metastasis, Malignant mixed müllerian tumor, Ovarian carcinosarcoma

## Abstract

Skin metastasis of ovarian cancer is extremely rare. We report an unusual case of ovarian carcinosarcoma with cutaneous metastasis of carcinomatous component that displayed distinct clinical manifestation. A 48-year-old woman presented to the dermatologist complaining of a new onset of erythematous, plaque-like skin rash with multiple small nodules on the left inner thigh, the area measuring 8 × 5cm. While the patient had no history of dermatologic conditions, she underwent a total hysterectomy and bilateral salpingo-oophorectomy, omentectomy, and lymph node dissection 16 months ago with a pathology confirmed stage IIIC ovarian carcinosarcoma. Of note, the carcinomatous component, mainly adenocarcinoma with hybrid features of seromucinous, endometrioid and minor high-grade serous carcinoma, involved bilateral fallopian tubes, omentum, and parametrium with extensive lymph node metastases. A skin biopsy specimen revealed an adenocarcinoma involving epidermis, dermis, and subcutaneous tissue with nodular contours, consistent with metastatic carcinomatous component of carcinosarcoma. Both carcinomatous component of primary ovarian carcinosarcoma and metastatic adenocarcinoma in the skin demonstrated Pax8, WT-1, and ER positivity and a mutation pattern of p53. The patient passed away 15 months after identification of skin metastasis. This case represents a unique example of cutaneous metastasis of ovarian carcinosarcoma with distinct clinical manifestation and detailed histopathological description. Alertness to the possibility of cutaneous metastasis, in combination with clinical history, morphological and immunohistochemical findings, is critical for a definitive classification.

## Introduction

Ovarian carcinosarcoma, also known as malignant mixed müllerian tumor (MMMT), is a biphasic neoplasm composed of high-grade malignant epithelial (carcinoma) and mesenchymal (sarcoma) elements, accounting for approximately 2% of all ovarian malignancies [1]. The malignant epithelial component is most commonly a high-grade serous carcinoma (HGSC) but can be of any of the surface epithelial types including clear cell and endometrioid carcinoma, or a carcinoma with mixed/hybrid morphology. The literatures have demonstrated that the carcinomatous and sarcomatous components are clonally related [2–5]; thus, these tumors should be best regarded as metaplastic carcinoma for which the sarcoma is considered as a result of epithelial-mesenchymal transition [6].

Carcinosarcomas are highly aggressive, fatal tumors with a median survival less than 24 months and the 5-year survival 15–30% [7]. Their stage distribution, behavior and patterns of spread are similar to that of HGSC. While intraperitoneal dissemination is considered the most common pattern of ovarian cancer spread, the tumor may also metastasize through the lymphatic channels and the hematogenous route to pleura, lung, liver, and lymph nodes [8]. Skin metastases are very rare, occurring in 0.9–5.8% of patients with ovarian cancer [8–11]. Here we report an unusual case of ovarian carcinosarcoma with cutaneous metastasis of carcinomatous component that displayed distinct clinical manifestation.

## Case study

### Clinical course

**Fig. 1:**
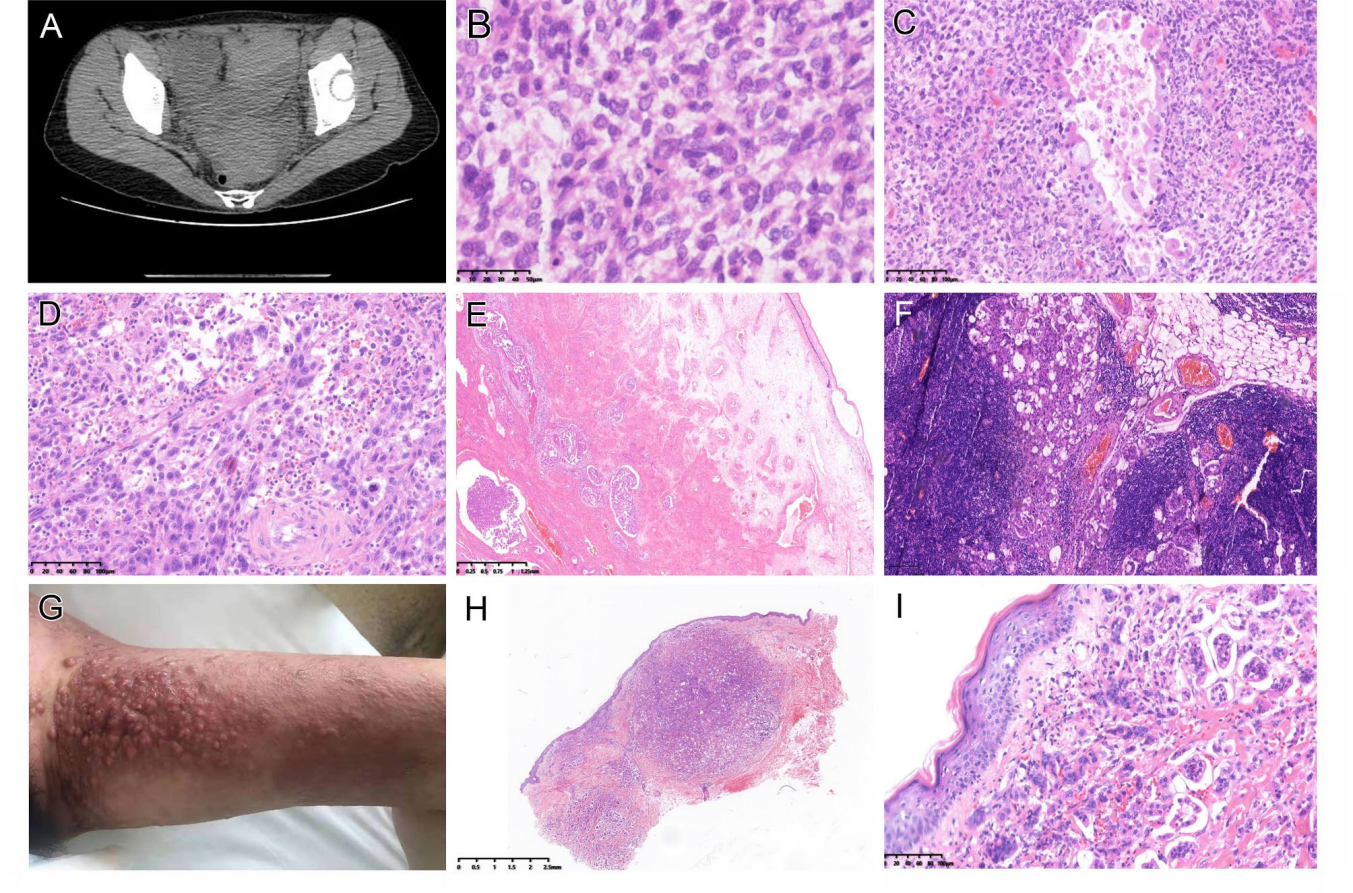
(A) Computed Tomography (CT) scan showed that the right ovarian mass extending to the left. The tumor displayed a biphasic morphology composed of intimately admixed carcinoma and sarcoma. (B) Predominant component was of homologous sarcoma. (C) Focal seromucinous/endometrioid carcinomatous component was present. (D) Focal area showed features of high-grade serous carcinoma. (E) The uterine cervix with extensive lymphovascular space invasion. (F) Pelvic lymph node with metastatic carcinoma. (G) 16 months after the initial diagnosis of ovarian carcinosarcoma, the patient presented with a skin lesion in the left inner thigh. (H) High grade adenocarcinoma involving epidermis, dermis, and subcutaneous tissue with nodular contour. (I) The morphology of skin tumor was similar to that of carcinomatous component of ovarian carcinosarcoma

The patient was a 48 year-old woman who presented with a right adnexal mass and extended to the left (Fig.1A) and subsequently underwent a total hysterectomy and bilateral salpingo-oophorectomy (TH/BSO), omentectomy, and lymph node dissection in October 2018. With a pathology confirmed stage IIIC ovarian carcinosarcoma, the patient received 4 cycles of chemotherapy. Two months later, ultrasound revealed a hypoechoic nodule behind the bladder, suspicious for tumor recurrence. Chemotherapy continued and a second debulking procedure was performed at 13 months after TH/BSO. The histopathological examination revealed highly atypical cells in a background of tumor necrosis, consistent with treatment effect. The patient received 5 more cycles of chemotherapy. In February 2020, the patient presented to the dermatologist complaining of a new onset of erythematous, plaque-like skin rash with multiple small nodules on the left inner thigh, measuring 8 × 5cm. She had no history of dermatologic conditions. A skin biopsy revealed metastatic carcinoma interpreted by the dermatopathologist with consultation from the gynecologic pathologist. The rash had progressively grown in size and some larger nodules were noted. A subsequent positron emission tomography/computed tomography (PET/CT) scan showed hypermetabolic signals in multiple lymph nodes, abdominal and pelvic cavity, retroperitoneum and left thigh, all indicating recurrence/metastasis. The patient passed away in May 2021. She had a history of myomectomy for fibroid in 2008 and fibroadenoma excision in the left breast in 2012, but without any significant family history.

## Gross and histopathological findings

Gross examination of TH/BSO specimen (2018) revealed right ovarian mass measuring 17cm in greatest dimension. Multiple tumor nodules were seen in the omentum with the largest one measuring > 2cm. Histologically, the tumor displayed a biphasic morphology composed of intimately admixed high-grade carcinoma and sarcoma (Fig.1B-D), with the vast majority (more than 80% of the ovarian tumor) being sarcomatous component (Fig.1B). The carcinomatous component exhibited morphology of seromucinous, endometrioid, and minor HGSC (Fig.1D) whereas the sarcomatous element was classified as homologous in that the stromal component had a non-specific appearance. The tumor, mainly the carcinomatous component, involved bilateral fallopian tubes, omentum, and parametrium with extensive lymphovascular space invasion (Fig.1E). Metastatic carcinoma was present in 30 of 37 pelvic, paraaortic, and omental lymph nodes (Fig.1F).

The skin lesion was located in the left inner thigh (Fig.1G). Microscopic examination of the skin biopsy specimen (2020) revealed an adenocarcinoma involving epidermis, dermis, and subcutaneous tissue with nodular contours (Fig.1H). Lymphovascular space invasion was present. The tumor displayed a morphology similar to that of carcinomatous component of ovarian carcinosarcoma (Fig.1I).

## Immunohistochemistry and BRCA testing

**Fig. 2:**
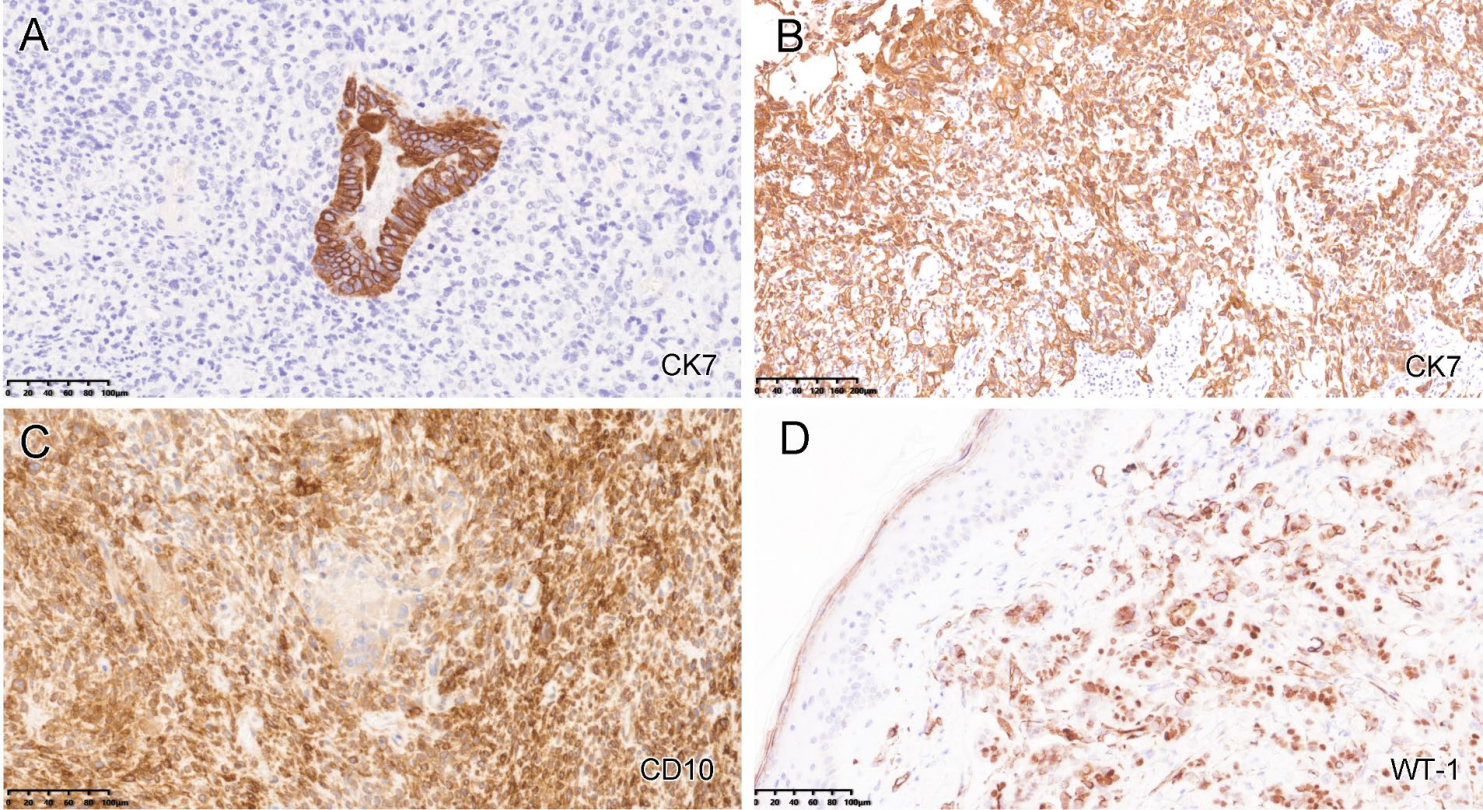
Immunohistochemical findings in ovarian carcinosarcoma and metastatic carcinoma in the skin. The carcinomatous component of carcinosarcoma, corresponding to Fig.1C and 1D, was diffusely positive for CK-7 (A and B), whereas the sarcomatous component was positive for CD10 (C). The metastatic carcinoma was positive for WT-1 (D)

**Fig. 3:**
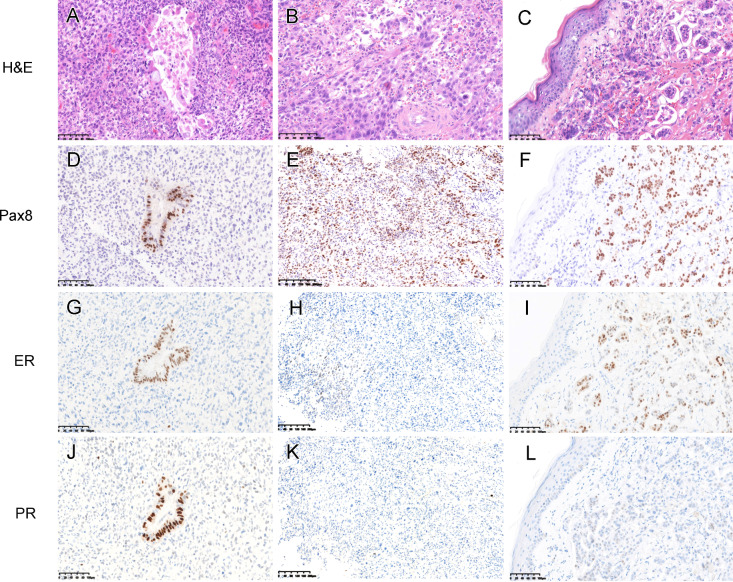
The carcinomatous component of ovarian carcinosarcoma (A and B, corresponding to Fig.1C and 1D) and skin metastatic adenocarcinoma (C, corresponding to Fig.1I) were positive for Pax8 (D-F). ER was diffusely positive in the seromucinous/endometrioid carcinoma (G) and metastatic carcinoma (I) but showed only weak and focal positivity in the high-grade serous carcinoma (H). PR was also diffusely positive in the seromucinous/endometrioid carcinoma (J) but negative in other component (K, L)

For the ovarian carcinosarcoma (2018), the seromucinous/endometrioid carcinomatous component was diffusely positive for CK-7 but the sarcomatous component was negative (Fig.2A). The HGSC component also displayed a diffuse CK7 staining (Fig.2B). The homologous sarcomatous component was positive for CD10 (Fig.2C) and vimentin. Both HGSC and metastatic carcinoma in the skin were positive for WT-1 (Fig.2D) with the former showing high-background staining. p53 displayed a possible “null” pattern mutation in both components (completely negative but lacking well-established internal control). p16 was negative in all different components. Corresponding to the areas of interest in Fig.1C, 1D, and 1I, the seromucinous/endometriod carcinoma (Fig.3A), HGSC (Fig.3B) and metastatic carcinoma (Fig.3C) were all positive for Pax8 (Figs. 3D-F). ER was diffusely positive in seromucinous/endometrioid carcinoma (Fig.3G) and metastatic carcinoma (Fig.3I) but weakly and focally positive in HGSC (Fig.3H). PR was only positive in seromucinous/endometrioid carcinoma (Fig.3J) but negative in the other two components (Fig.3K, 3L). The morphology and immunoprofile support that the metastatic carcinoma in the skin originated from the carcinomatous component of ovarian carcinosarcoma.

Targeted analysis of BRCA1 and BRCA2 mutations was performed using genomic DNA extracted from formalin-fixed paraffin embedded (FFPE) tumor tissue. No pathogenic BRCA mutations were found in the primary or metastatic tumors.

## Discussion

This case represents a unique example of cutaneous metastasis of ovarian carcinosarcoma with distinct clinical manifestation and detailed histopathological description. The most perplexing aspect of this case was the patient’s clinical presentation with a skin rash on the inner thigh which was evaluated by a dermatologist. Since the patient had a known history of ovarian carcinosarcoma and had been initially treated with paclitaxel and carboplatin, and then switched to docetaxel/cisplatin/bevacizumab, chemotherapy-related drug reaction was first considered as the possible cause. In fact, it has been reported that dermatological toxicities, from diffuse macules and papules to rarely life-threatening erythema multiforme, toxic epidermal necrolysis, and Stevens-Johnson syndrome, clearly represent one of the major adverse events associated with these agents [12–14]. In addition, inner thigh is an anatomic site which is easily irritated by friction, allowing bacteria and other germs to grow and causing skin rash. With these considerations, a skin biopsy facilitated an unexpected diagnosis of tumor metastasis.

Given the ovary’s anatomic location, it is understandable that the most common route of ovarian cancer spread is intraperitoneal dissemination and intra-abdominal pelvic and para-aortic lymphatic metastases. Although rare, cutaneous metastasis of ovarian cancer has been well documented. Two large case series studies revealed that 3.3% (10 of 300) and 4% (10 of 242) of women with skin metastasis were derived from primary ovarian tumor [15, 16]. On the other hand, different studies reported variable rate of ovarian cancer with cutaneous metastases. In one study, 9 (4%) of 220 ovarian cancer patients had skin metastases [10], whereas another study reported that the cutaneous metastasis rate was up to 5.8% (12 of 206) [9]. In general, skin metastases can be classified as Sister Mary Joseph’s nodule (SJN, metastatic umbilical tumor) and non-SJN skin metastases [17]. While most of the former are identified at the time of initial diagnosis, the latter usually develop in recurrent settings. Mechanistically, contiguous extension and lymphatic spread are major modes in the development of SJNs. In contrast, surgical site implantation, extranodular extension, and hematogenous spread may be involved in non-SJN metastases. In our case, the patient had a stage IIIC disease with extensive lymph node metastases at the time of TH/BSO. We suspect the skin involvement, presenting as non-SJN metastasis in this case, resulted from lymphatic or hematogenous spread during the course of the disease. In fact, the presence of lymphovascular space invasion in the dermis and subcutaneous tissue provides strong evidence to support this speculation. Our patient also presented with hypermetabolic signals in the bilateral inguinal regions when a PET/CT scan was performed. Although lymph node in these regions was not biopsied and no pathology was confirmed, we suspect lymphatic spread with extra-nodular extension is the most likely alternative path of spread, although this is largely speculative.

Another unique aspect of this case is its histopathological type. Ovarian carcinosarcoma is a biphasic, high-grade malignant neoplasm which commonly affects the patients older than 60 years and often is of high stage at the time of diagnosis [1]. Given the patient’s young age (48 year-old) and the close relation between carcinosarcoma and a minor HGSC component in the ovary, *BRCA1/2* mutation was tested. Although no germline or somatic *BRCA* mutation was detected, whether there are genetic alterations in other susceptible genes including *PALB2*, *ATM*, *CHEK2*, and *RAD51*, is not known.

Carcinosarcomas are thought to be of epithelial origin with molecular studies showing identical *TP53* mutations in the carcinomatous and sarcomatous components [2, 4, 5]. Like its ovarian counterpart, a study of 40 cases of uterine carcinosarcoma with metastasis demonstrated that 75% (30 of 40) of metastatic tumors were purely carcinoma [18]. Although the presence of metastatic sarcoma is thought to be an adverse prognostic factor [19], the metastases and recurrences of ovarian carcinosarcoma is usually HGSC which is considered as the main driving force behind metastatic disease. Consistently, the main tumor in our case was ovarian carcinosarcoma and most of metastatic lesions were carcinoma at the time of the diagnosis. The cutaneous metastasis during the course of the disease was also confirmed as an adenocarcinoma which is consistent with the carcinomatous component of carcinosarcoma.

While cutaneous metastasis of primary ovarian carcinomas, including HGSC, endometrioid carcinoma and clear cell carcinoma, has been well described in the literature [9, 10, 20–22], our case represents a unique example of ovarian carcinosarcoma with distant skin metastasis. Considering its uterine counterpart, several case studies of uterine carcinosarcoma with cutaneous metastasis have been reported [23, 24]. A recent case study reported a 57-year-old woman with multiple subcutaneous nodules on the face and trunk with pathology confirmed as the sarcomatous component of the primary uterine carcinosarcoma [24]. This patient, unlike our case, presented with multiple subcutaneous nodules on the face and trunk rather than skin rash. In fact, skin metastases can take on different clinical manifestations including isolated or multiple cutaneous nodules, macules and papules, sclerotic plaques, and inflammatory changes [17].

Since cutaneous metastasis is extremely rare, differential diagnosis should always be considered in spite of a history of malignancy. These include the chemotherapy-related drug reaction, as we discussed above, non-malignant dermatological diseases such as eczema, psoriasis, pyogenic granuloma and hypertrophic scar, and skin neoplastic processes like Paget disease and primary adnexal tumor [17]. In our case, the tumor in the epidermis and dermis displayed a similar morphology to that of HGSC component of ovarian carcinosarcoma. A panel of immunomarkers including Pax8, WT-1, p53, ER, and PR facilitated the diagnosis. In particular, it has been demonstrated that Pax8 is a useful marker that effectively discriminated metastatic ovarian carcinomas from metastatic breast carcinomas and primary adnexal tumors [25].

As a stage IV disease, it is not surprising that the patients with ovarian cancer developing skin metastases have a poor prognosis comparable to the disease in other stage IV locations [17]. However, the prognosis of cutaneous metastases in ovarian cancer varies due to many affecting factors. The patients with SJN or skin metastases occurring in surgical scars of primary procedure may have a relatively favorable prognosis [9]. In contrast, the patients with a non-SJN skin lesion rather than previous surgical site have a poor prognosis as this type of cutaneous metastasis usually develops during the course of disease progression when multiple metastases occur. The skin metastasis in our patient developed at 16-month after the initial diagnosis. She passed away at 15-month after the diagnosis of skin metastasis.

In summary, we report a rare case of ovarian carcinosarcoma with cutaneous metastasis of the carcinomatous component. Alertness to the possibility of cutaneous metastasis, in combination with clinical history, morphological and immunohistochemical findings, is critical for a definitive classification.

## Abbreviations

FFPE: Formalin-fixed paraffin-embedded.
HGSC: high grade serous carcinoma.
MMMT: malignant mixed müllerian tumor.
PET/CT: positron emission tomography/computed tomography.
SJN: Sister Mary Joseph’s nodule.
TH/BSO: total hysterectomy and bilateral salpingo-oophorectomy.

## References

[1] Kurman RJ, Carcangiu ML, Herrington CS (2014). WHO Classification of Tumors of Female Reproductive Organs.

[2] Fujii H, Yoshida M, Gong ZX (2000). Frequent genetic heterogeneity in the clonal evolution of gynecological carcinosarcoma and its influence on phenotypic diversity. Cancer Res Jan.

[3] Gallardo A, Matias-Guiu X, Lagarda H (2002). Malignant mullerian mixed tumor arising from ovarian serous carcinoma: a clinicopathologic and molecular study of two cases. Int J Gynecol Pathol Jul.

[4] Jin Z, Ogata S, Tamura G (2003). Carcinosarcomas (malignant mullerian mixed tumors) of the uterus and ovary: a genetic study with special reference to histogenesis. Int J Gynecol Pathol Oct.

[5] Abeln EC, Smit VT, Wessels JW (1997). Molecular genetic evidence for the conversion hypothesis of the origin of malignant mixed mullerian tumours. J Pathol Dec.

[6] Zhao S, Bellone S, Lopez S (2016). Mutational landscape of uterine and ovarian carcinosarcomas implicates histone genes in epithelial-mesenchymal transition. Proc Natl Acad Sci U S A Oct.

[7] Rauh-Hain JA, Growdon WB, Rodriguez N (2011). Carcinosarcoma of the ovary: a case-control study. Gynecol Oncol Jun.

[8] Dauplat J, Hacker NF, Nieberg RK (1987). Distant metastases in epithelial ovarian carcinoma. Cancer Oct.

[9] Otsuka I, Matsuura T (2017). Skin metastases in epithelial ovarian and fallopian tube carcinoma. Med (Baltimore) Aug.

[10] Cormio G, Capotorto M, Di Vagno G (2003). Skin metastases in ovarian carcinoma: a report of nine cases and a review of the literature. Gynecol Oncol Sep.

[11] Cheng B, Lu W, Xiaoyun W (2009). Extra-abdominal metastases from epithelial ovarian carcinoma: an analysis of 20 cases. Int J Gynecol Cancer May.

[12] Sibaud V, Leboeuf NR, Roche H (2016). Dermatological adverse events with taxane chemotherapy. Eur J Dermatol Oct.

[13] Caiado J, Picard M (2014). Diagnostic tools for hypersensitivity to platinum drugs and taxanes: skin testing, specific IgE, and mast cell/basophil mediators. Curr Allergy Asthma Rep Aug.

[14] Holynska-Iwan I, Sobiesiak M (2020). Cisplatin influences the skin ion transport - An in vitro study. Biomed Pharmacother Sep.

[15] Lookingbill DP, Spangler N, Helm KF (1993). Cutaneous metastases in patients with metastatic carcinoma: a retrospective study of 4020 patients. J Am Acad Dermatol Aug.

[16] Brownstein MH, Helwig EB (1972). Metastatic tumors of the skin. Cancer May.

[17] Otsuka I (2019). Cutaneous Metastases in Ovarian Cancer. Cancers (Basel) Sep.

[18] Silverberg SG, Major FJ, Blessing JA (1990). Carcinosarcoma (malignant mixed mesodermal tumor) of the uterus. A Gynecologic Oncology Group pathologic study of 203 cases. Int J Gynecol Pathol.

[19] Kunkel J, Peng Y, Tao Y (2012). Presence of a sarcomatous component outside the ovary is an adverse prognostic factor for primary ovarian malignant mixed mesodermal/mullerian tumors: a clinicopathologic study of 47 cases. Am J Surg Pathol Jun.

[20] Wiechert AC, Garrett LA, Lin G (2012). Management of a skin metastasis in a patient with advanced ovarian cancer. Gynecol Oncol Case Rep.

[21] Husein-ElAhmed H, Aneiros-Fernandez J, Arias-Santiago S (2010). Sister Mary Joseph’s nodule as a metastasis of ovarian adenocarcinoma. Int J Dermatol Sep.

[22] Nie X, Chen X, Jiang Y (2022). Sister Mary Joseph nodule as cutaneous manifestations of metastatic ovarian cancer: A case report and review of the literature. Med (Baltimore) Feb.

[23] Callister M, Ramondetta LM, Jhingran A (2004). Malignant mixed Mullerian tumors of the uterus: analysis of patterns of failure, prognostic factors, and treatment outcome. Int J Radiat Oncol Biol Phys Mar.

[24] Clairwood M, Yasuda M, Belazarian L (2016). Unusual Cutaneous Metastasis of Uterine Carcinosarcoma: A Case Report and Review of the Literature. Am J Dermatopathol May.

[25] Fujiwara M, Taube J, Sharma M (2010). PAX8 discriminates ovarian metastases from adnexal tumors and other cutaneous metastases. J Cutan Pathol Sep.

